# Data on spatiotemporal land use land cover changes in peri-urban West Arsi Zone, Ethiopia: Empirical evidences from Shashemene peri-urban areas

**DOI:** 10.1016/j.dib.2018.03.082

**Published:** 2018-03-22

**Authors:** Tarekegn Girma, Tebarek Lika, Molla Maru

**Affiliations:** a College of Social Science, Addis Ababa University, Ethiopia; b Department of Geography and Environmental Studies, Addis Ababa University, Ethiopia

## Abstract

Urban expansion is one of the major problems in Ethiopia resulting in displacement of the rural people inhabiting areas bordering the cities/towns. It is also resulting in land use land cover (LULC) changes affecting the livelihoods of the people and the ecosystems (Messay et al., 2017; Ganamo, 2013) [[1], [2]]. The data presented in this article, therefore shows the spatiotemporal LULC changes of peri-urban expansion areas of Shashemene City. The data were generated from Landsat Thematic Mapper (TM) and Enhanced Thematic Mapper plus (ETM^+^) images (with path/row numbers 168/055) from EarthExplorer.usgs.gow and the data was classified, interpreted and cross-tabulated using ERDAS IMAGIN 2013 and ArcGIS 10.4.1 software packages. The accuracy of the image classification was verified by geo-location data collected from ground control points by using Geo Positioning System (GPS) receiver and the spatial resolution (1 m) and very recent (2016) Imagery downloaded from Google Earth. The result indicates that the built-up areas have increased by 1938.71 ha (19.3871 km^2^) with 73.4%, and 17.6% decline in forest land and grassland respectively between 1973 and 2016.

**Specifications Table**

**Table:** 

Subject area	Geography and Environmental Studies
More specific	
Subject area	Land use land cover change, urban sprawl
Type of data	Table, figure and text file
How data was acquired	Data were extracted from Landsat TM and Landsat ETM^+^ images with path/row numbers 168/055 and first hand data were acquired by using GPS-based ground survey technique.
Data format	Analyzed
Experimental factors	
Experimental features	The images were geo-referenced with World Geodetic System (WGS) 1984 datum and Universal Transverse Mercator (UTM) projection system zone 37North. The images were classified based on visual interpretation and supervised classification using ERDAS IMAGINE 2013 and ArcGIS 10.4.1 software packages.
Data source location	Landsat and Shashemene area (7° 9′5″N - 7° 18′17″N, 38° 31′43″E - 38° 41′58″E)
Data accessibility	The data is with this article.

**Value of the Data**

• The data is helpful to Shashemene City municipality to venture the extent of the spatiotemporal expansion of Shashemene and its potential effect on the City periphery.
• The data provides information on the status of urban expansion towards rural peri-urban areas around Shashemene.
• The data is vital to model urban expansion towards rural peri-urban areas surrounding Shashemene to mitigate its adverse effect on the livelihoods of the people inhibiting the area and the eco system.
• The data is useful to researchers, urban planners and experts working in the field.

## 1 Data

**Fig. 1:**
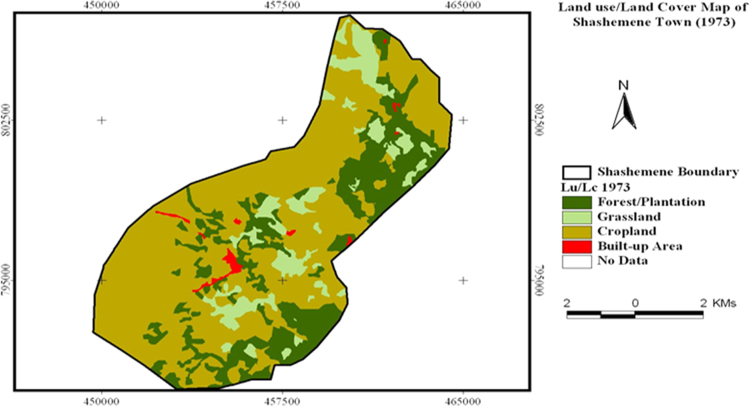
LULC classes of Shashemene urban areas in 1973.

**Fig. 2:**
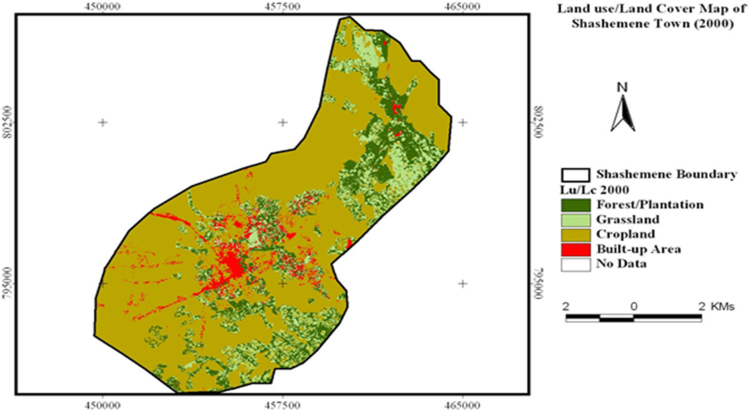
LULC classes of Shashemene urban areas in 2000.

**Fig. 3:**
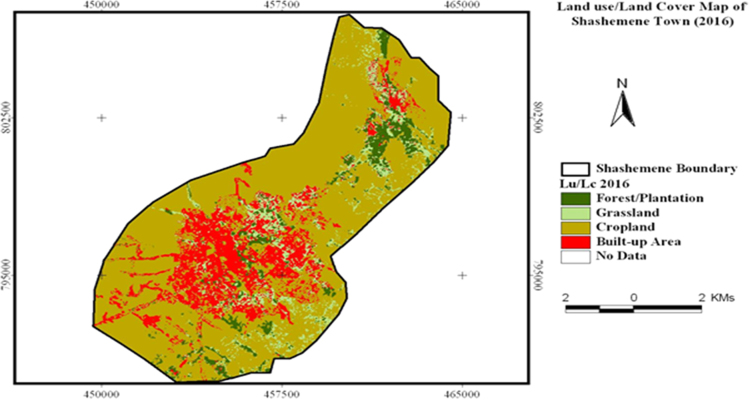
LULC classes of Shashemene urban areas in 2016.

**Fig. 4:**
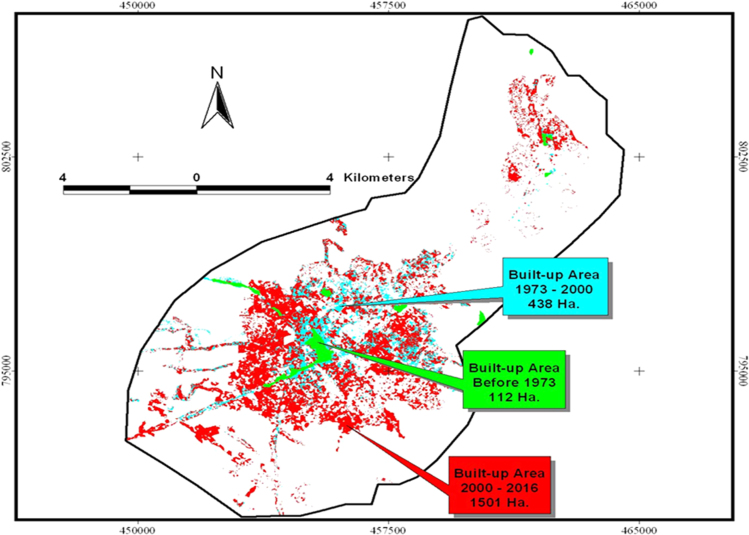
Rate of LULC change in hectare per year.

The data in this article offers information on the spatiotemporal LULC changes in Shashemene urban expansion areas between 1973 and 2016. Fig. 1, Fig. 2, Fig. 3 illustrate pictorially the spatiotemporal LULC classes of the area in 1973, 2000 and 2016. Crop land and grass land had dominated the land use in 1973 (Fig. 1) with very few built up areas, plantation and forestland. In 2000 (Fig. 2), plantation was tremendously expanded and crop land was considerably reduced. In 2016 (Fig. 3), built up area was extremely enlarged, crop land was almost disappeared and some part of the cropland was replaced by built up areas. Table 1 demonstrates LULC extent in hectare and percentage in 1973, 2000 and 2016 as well as rate of LULC changes in hectare (also Fig. 4) and percentage (%). Table 2, Table 3, Table 4 demonstrate LULC change matrix between 1973 and 2000, 2000 and 2016, and 1973 and 2016.

**Table 1:** LULC extents and changes in Shashemene urban areas (1973–2016).

LULC classes	1973		2000		2016		Change 1973–2000		Change 2000–2016		Change 1973–2016	
	ha	%	ha	%	ha	%	ha	%	ha	%	ha	%
Forest land/Plantation	3007.8	23.990	1996.3	15.923	798.9	6.372	−1011.4	−33.6	−1197.5	−60.0	−2208.9	−73.4
Grassland	1301.4	10.4	1633.6	13.0	1071.9	8.5	332.2	25.5	−561.7	−34.4	−229.5	−17.6
Cropland	8114.0	64.7	8355.2	66.6	8613.8	68.7	241.2	3.0	258.547	3.1	499.725	6.2
Built up	114.4	0.9	552.5	4.4	2053.1	16.4	438.1	382.9	1500.64	271.6	1938.71	1694.5
Total	12,537.6	100.0	12,537.6	100.0	12,537.6	100.0	0.0		0.0		0.0

**Table 2:** Land use/Land cover change matrix in Shashemene Town during the period 1973–2000.

	Lu/Lc type	Forest/Plantation		Grassland		Cropland		Built-up Area		Total	
		Ha.	%	Ha.	%	Ha.	%	Ha.	%	Ha.	%
Land use/Land cover Types 2000	Forest/Plantation	1483.0	49.3	513.3	39.4	0.0	0.0	0.0	0.0	1996.3	15.9
	Grassland	1057.6	35.2	576.0	44.3	0.0	0.0	0.0	0.0	1633.6	13.0
	Cropland	367.9	12.2	130.9	10.1	7856.5	96.8	0.0	0.0	8355.2	66.6
	Built-up Area	99.2	3.3	81.3	6.2	257.6	3.2	114.4	100.0	552.5	4.4
	Total	3007.8	100.0	1301.4	100.0	8114.0	100.0	114.4	100.0	12,537.6	100.0

**Table 3:** Land use/Land cover change matrix in Shashemene Town during the period 2000–2016.

	Lu/Lc type	Forest/Plantation		Grassland		Cropland		Built-up Area		Total	
		Ha.	%	Ha.	%	Ha.	%	Ha.	%	Ha.	%
Land use/Land cover Types 2016	Forest/Plantation	539.8	17.9	139.5	10.7	57.9	0.7	61.6	53.8	798.9	6.4
	Grassland	562.4	18.7	389.0	29.9	0.0	0.0	120.5	105.3	1071.9	8.5
	Cropland	592.5	19.7	869.8	66.8	7132.2	87.9	19.2	16.8	8613.8	68.7
	Built-up Area	301.6	10.0	235.3	18.1	1165.1	14.4	351.2	306.9	2053.1	16.4
	Total	1996.3	66.4	1634	125.5	8355.2	103.0	552.5	482.9	12,537.6	100.0

**Table 4:** Land use/Land cover change matrix in Shashemene Town during the period 1973–2016.

	Lu/Lc type	Forest/Plantation		Grassland		Cropland		Built-up Area		Total	
		Ha.	%	Ha.	%	Ha.	%	Ha.	%	Ha.	%
Land use/Land cover Types 2016	Forest/Plantation	547.9	18.2	152.4	11.7	86.8	1.1	11.7	10.2	798.9	6.4
	Grassland	662.0	22.0	346.4	26.6	52.6	0.6	10.9	9.5	1071.9	8.5
	Cropland	1410.7	46.9	500.4	38.4	6691.1	82.5	11.6	10.1	8613.8	68.7
	Built-up Area	387.1	12.9	302.2	23.2	1283.6	15.8	80.2	70.1	2053.1	16.4
	Total	3007.8	100.0	1301.4	100.0	8114.0	100.0	114.4	100.0	12,537.6	100.0

## 2 Experimental design, materials and methods

Landsat Thematic Mapper (TM) and Enhanced Thematic Mapper plus (ETM+) images (with path/row numbers 168/055) as well as GPS-based ground survey records were vigorous data sources for this data article. The analysis, such as data extraction and LULC classification, interpretation and creation of change matrices were done by using ERDAS IMAGINE version 2013 and ArcGIS 10.4.1 software. The images were geo-referenced with World Geodetic System (WGS) 1984.

datum and Universal Transverse Mercator (UTM) projection system zone 37 North. Supervised and unsupervised image classifications techniques were applied to extract the data [3]. Supervised classification involved selecting pixels that represent land cover classes that are documented by the expert. Unsupervised image classification is computer- automated. It allows the expert to specify some parameters that the computer uses to disclose statistical patterns that are intrinsic in the data. These patterns are bands of pixels with similar spectral features. Due to similar spectral appearances of grass, and crop, which were determined to be independent classes before classification, the application of unsupervised classification might not provide decent results. As a result, in the data extraction process, supervised image classification was used. After defining the land use features, the next step was deriving LULC change matrices. This was done through overlaying the classified satellite images and analyzing by image differencing algorithm. Lastly, the outputs of images classification were verified by conducting ground truth while recoding *x* and *y* co-ordinates of sample spatial features using GPS. Based on the scope of the study and pictorial interpretation of the satellite imageries, four classes were identified in Shashemene urban areas. These are Forest land/ Plantation, Grassland, Cropland and Built up in the vicinity.
