# Supplementary material for: Data on spatiotemporal land use land cover changes in peri-urban West Arsi Zone, Ethiopia: Empirical evidences from Shashemene peri-urban areas
Source: Data Brief. 2018 Mar 22;18:747–52. doi: 10.1016/j.dib.2018.03.082 (PMC5996349; doi:10.1016/j.dib.2018.03.082)
Supplement: Supplementary file 1 — Supplementary material [file mmc1.docx]

**Conflict of Interest and Authorship Conformation Form** 
Please check the following as appropriate:
 
All authors have participated in (a) conception and design, or analysis and interpretation of the data; (b) drafting the article or revising it critically for important intellectual content; and (c) approval of the final version. **YES**
 
This manuscript has not been submitted to, nor is under review at, another journal or other publishing venue. **YES**
 
The authors have no affiliation with any organization with a direct or indirect financial interest in the subject matter discussed in the manuscript. **YES**
